# Supplementary figures and images for: Moraxella-dominated pediatric nasopharyngeal microbiota associate with upper respiratory infection and sinusitis
Source: PLoS One. 2021 Dec 28;16(12):e0261179. doi: 10.1371/journal.pone.0261179 (PMC8714118; doi:10.1371/journal.pone.0261179)

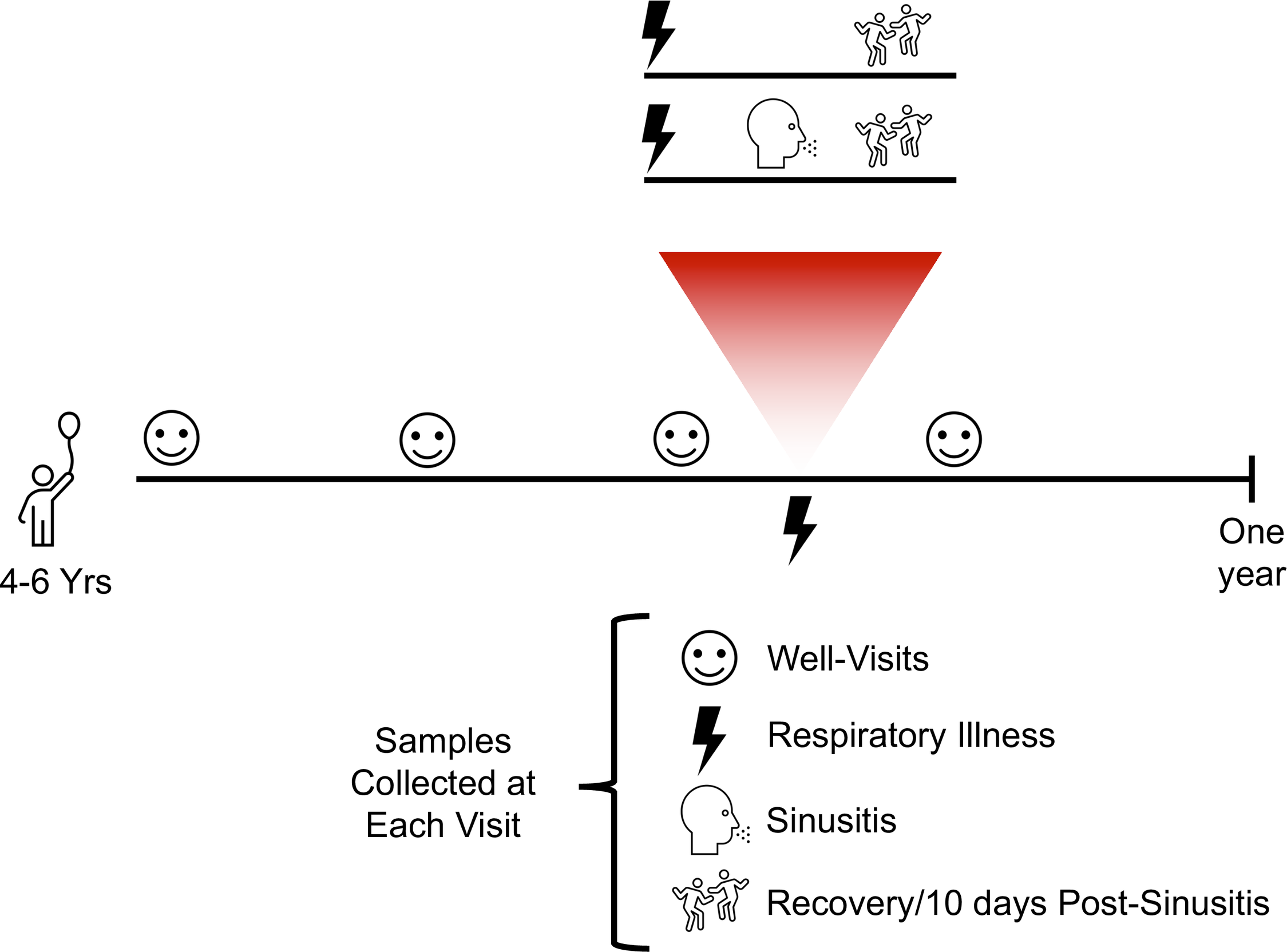

Supplement: S1 Fig — (TIF) [file pone.0261179.s001.tif]

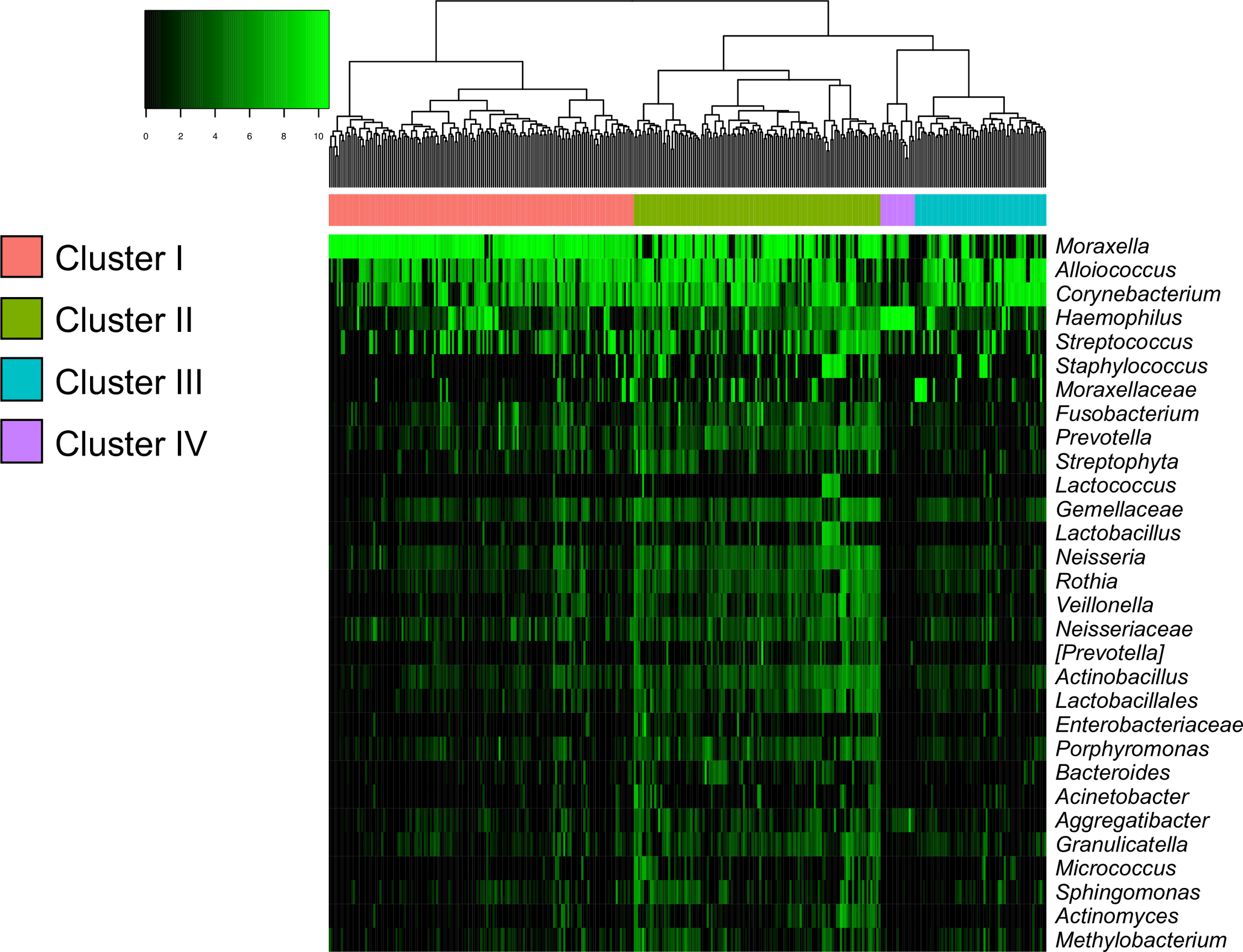

Supplement: S2 Fig — Taxa were agglomerated at the genus level, and abundances were log-transformed. The top 30 genera are presented in the heatmap. (TIF) [file pone.0261179.s002.tif]
